# Characteristics of Hospitalized COVID-19 Patients at Admission and Factors Associated with Clinical Severity in Low- and Middle-Income Countries: An Observational Study

**DOI:** 10.4269/ajtmh.23-0456

**Published:** 2024-02-27

**Authors:** Marianne Abifadel, Kaousar Ahmmed, Sayera Banu, Ibrahima Camara, Fahmida Chowdhury, Daouda Coulibaly, Georges Dabar, Cédric Dananché, Rachel Daw, Zakiul Hassan, Magali Hervé, Ariful Islam, Florence Komurian-Pradel, Jean-Pierre Kouamé, Bourema Kouriba, Josette Najjar-Pellet, Andoniaina Rakotonaivo, Felana Ranaivo-Rabetokotany, Mandranto Rasamoelina, Tiavina Rasolofoarison, Moussa Riachi, Mitra Saadatian-Elahi, Luc Samison, Valentina Sanchez Picot, Sita Savané, Ismaila Thera, Abdoulaye Touré, Philippe Vanhems

**Affiliations:** ^1^ Laboratoire Rodolphe Mérieux, Université Saint-Joseph, Beirut, Lebanon;; ^2^ International Centre for Diarrhoeal Disease Research, Bangladesh (icddr,b), Dhaka, Bangladesh;; ^3^ Health Emergencies Program, World Health Organization, Conakry, Guinea;; ^4^ Institut National d’Hygiène Publique, Abidjan, Ivory Coast;; ^5^ Hôpital Hôtel Dieu de France, Alfred Naccache Boulevard, Beirut, Lebanon;; ^6^ Service Hygiène, Epidémiologie, Infectiovigilance et Prévention, Hospices Civils de Lyon, France;; ^7^ Centre International de Recherche en Infectiologie, Public Health, Epidemiology and Evolutionary Ecology of Infectious Diseases Team, Institut National de la Santé et de la Recherche Médicale U1111, Centre National de la Recherche Scientifique Unité Mixte de Recherche 5308, École Nationale Supérieure de Lyon, Université Claude Bernard Lyon 1, France;; ^8^ Fondation Mérieux, Lyon, France;; ^9^ Hospices Civils de Lyon, Pôle Santé Publique, Bases Cliniques—Epidémiologiques, Service Hospitalo-Universitaire de Pharmacotoxicologie, Lyon, France;; ^10^ Centre d’Infectiologie Charles Mérieux, Bamako, Mali;; ^11^ Fondation Mérieux, Beirut, Lebanon;; ^12^ Centre d’Infectiologie Charles Mérieux, Ankatso, Antananarivo, Madagascar;; ^13^ Centre International pour l’Excellence dans la Recherche (ICER), Bamako, Mali;; ^14^ Institut National de Santé Publique, Ministère de la Santé, Conakry, Guinea

## Abstract

Despite the numerous articles published on the clinical characteristics and outcomes of COVID-19 with regard to high-income countries, little is known about patients in low- and middle-income countries (LMIC) in this context. The objective of this observational, prospective, hospital-based multicentric study was to describe clinical features and outcomes of laboratory-confirmed COVID-19 patients hospitalized in each of the participating centers in Bangladesh, Guinea, Ivory Coast, Lebanon, Madagascar, and Mali during the first year of the pandemic (March 5, 2020 to May 4, 2021). The study outcome was the clinical severity of COVID-19, defined as hospitalization in intensive care unit or death. Multivariate logistic regression models were performed to identify independent variables associated with disease severity. Overall, 1,096 patients were included. The median age was 49.0 years, ranging from 38.0 in Mali to 63.0 years in Guinea. The overall clinical severity of COVID-19 was 12.3%, ranging from 6.4% in Mali to 18.8% in Guinea. In both groups of patients <60 and ≥60 years old, cardiovascular diseases (adjusted odds ratio [aOR]: 1.99; 95% CI: 1.13–3.50, *P* = 0.02; aOR: 2.47; 95% CI: 1.33–4.57, *P* = 0.004) were independently associated with clinical severity, whereas in patients <60 years, diabetes (aOR: 2.13; 95% CI: 1.11–4.10, *P* = 0.02) was also associated with clinical severity. Our findings suggest that COVID-19-related severity and death in LMICs are mainly driven by older age. However, the presence of chronic diseases can also increase the risk of severity especially in younger patients.

## INTRODUCTION

The SARS-CoV-2 emerged in December 2019 in Wuhan, China and disseminated quickly throughout the world. As of March 10, 2023, a total of 676,609,955 of COVID-19–positive cases including 6,881,955 deaths had been reported worldwide.^1^ The WHO declared the end of COVID-19 global emergency on May 5, 2023. However, the virus continues to circulate despite massive immunization programs worldwide.

As for other infectious diseases, the pattern of COVID-19 in terms of symptoms, recovery, and mortality rates can be driven by the so-called epidemiologic triangle—that is, pathogen, host, and environment.^2^ Among host-related factors, older age and the presence of underlying comorbidities are known to be independent risk factors of COVID-19 severity and mortality.^3^ The age structure of the population in low- and middle-income countries (LMICs), such as most West African and South Asian countries, is mainly made up of young adults.^4^ As a consequence, one could hypothesize milder COVID-19 disease and lower rates of mortality in these countries. On the other hand, higher frequency of comorbidities, even in younger adults, in LMICs may increase the risk of severe disease.^5^^–^^7^

Overcrowded housing,^8^ co-circulation of myriad other infectious agents,^9^ and a weaker health system that could suffer from a lack of adequate tertiary care services equipped with intensive care units (ICUs)^10^ could also affect the course of the disease in LMICs.

Despite the numerous articles published with regard to high-income countries, little is known about the clinical characteristics and outcome of COVID-19 patients in LMICs. The aim of this study was to describe clinical features and outcomes of laboratory confirmed COVID-19 hospitalized patients consulting one of the participating centers in six LMICs during the first year of the SARS-CoV-2 pandemic.

## MATERIALS AND METHODS

### Study population.

NOSO-COR is an international observational, prospective, hospital-based multicentric study that has been carried out in 13 French hospitals and 17 centers in four West African countries (Guinea, two centers; Ivory Coast, five centers; Madagascar, three centers; and Mali, two centers), one South Asian country (Bangladesh, three centers), one Middle Eastern country (Lebanon, one center), and one South American country (Brazil, one center) (Supplemental Figure 1).^11^ Both patients and health care professionals (HCPs) presenting symptoms of COVID-19 were eligible to participate. The present work was limited to patients who had been hospitalized in six countries (16 centers). Brazil was excluded from the present analysis because the country had only enrolled HCPs. Patient enrollment occurred from March 5, 2020 (Lebanon) to May 30, 2020 (Guinea) and ended on May 4, 2021 in all centers. The start date of the inclusion period was not the same in all centers (see details in Supplemental Table 1) due to logistical issues related to the ethical committee approval and the availability of laboratory tests.

### Study outcome and follow-up.

The study outcome was COVID-19 clinical severity, defined as hospitalization in ICU or death. Hospitalized SARS-CoV-2 laboratory-confirmed patients were then provided with follow-up until hospital discharge or death.

### Data collection and study variables.

Data collection procedures and inclusion and exclusion criteria have been described elsewhere.^11^ Briefly, adult patients who presented an infectious syndrome based on the WHO case definition of COVID-19 as of March 30, 2020^12^ and consented to participate were eligible to participate. Suspected cases underwent nasopharyngeal swab collection using Flexible Minitip Flocked Swab with 3-mL UTM^®^ Viral Transport Medium (COPAN Diagnostics Inc., Brescia, Italy). Symptomatic patients with positive real-time reverse transcriptase polymerase chain reaction (RT-PCR) results were defined as confirmed cases of SARS-CoV-2 infection.

Demographic characteristics, underlying comorbidities, and clinical symptoms at hospital admission were collected using a standardized data collection form. Lung x-ray results were recorded when available. Major symptoms were defined as at least one symptom among the following: cough, fever, weakness, dyspnea, pain, and confusion. Minor symptoms were defined as at least one symptom among the following: headache, anosmia, ageusia, sore throat, runny nose, diarrhea, conjunctivitis, or without any major symptom. Any further complications that occurred during hospitalization were retrieved from patient medical files, up to hospital discharge or death.

General data on population demography per country, number of COVID-19 cases and deaths, vaccination situation, and the most predominant circulating strains during the study period were also collected.

## STATISTICAL ANALYSES

Because of the relatively small number of patients in each center, analyses were carried out by country. In accordance with the main objective of the study, that is, the risk of severe COVID-19, outpatients and patients with negative RT-PCR test, were excluded from the final analysis.

It is known that age is a risk factor of COVID-19 severity. We therefore carried out additional analysis by stratifying the population into two age groups: young patients (<60 years old) and older patients (≥60 years old) has also been performed. The threshold of 60 years old was based on the WHO definition of the elderly.

Continuous variables were reported as median and interquartile range (IQR) with comparisons using the Mann–Whitney *U* test. Qualitative variables were computed as number of individuals (*n*) and frequency (%) using the χ^2^ or Fisher exact test as appropriate for comparison.

Two multivariate logistic regression models were performed to identify independent variables associated with clinical severity in patients ≥60 and <60 years old. Gender was always included in the models. Underlying comorbidities (cardiovascular disease, diabetes, chronic lung disease, renal disease, and liver disease) associated with disease severity were retained in the model if *P* <0.20 in univariate analysis. Interactions between variables were tested. Hosmer–Lemeshow tests were used to assess model quality. All tests were two-tailed, with *P* <0.05 considered statistically significant. Statistical analysis was performed using STATA 17^®^ (College Station, TX).

## RESULTS

### COVID-19 context in participating countries.

Demographic characteristics and some general information about the context of COVID-19 in participating countries during the study period are reported in Table 1. The estimated median age was younger than 30.0 years in all countries, ranging from 15 years in Mali to 28 years in Lebanon. During the study period, Bangladesh reported the highest number of cases and deaths. COVID-19 vaccination programs began in Bangladesh, Guinea, and Lebanon during the study period, with Lebanon having the highest (3.3%) rates of vaccine coverage by the end of the study on May, 4, 2021. The most predominant circulating strains during the study period were the wild strain, followed by Alpha in Bangladesh, Guinea, Ivory Coast, and Lebanon. In Madagascar, the second most frequent strain was Beta. In Mali, both Alpha (20A/C)^13^ and Eta^14^ were present during the study period.

**Table 1:** Demographic characteristics and COVID-19 generalities in participating countries during the study period

Demographics	Bangladesh	Guinea	Ivory Coast	Lebanon	Madagascar	Mali
Estimated population, Jan. 1, 2021*	168,415,000	13,369,000	27,186,000	5,631,000	28,571,000	21,561,000
Estimated median age (years), Jan. 1, 2021*	26.3	17.7	17.6	28.3	19.0	15.1
Date of the first diagnosed COVID-19 case	Mar. 8, 2020	Mar. 13, 2020	Mar. 11, 2020	Feb. 21, 2020	Mar. 20, 2020	Mar. 25, 2020
Cumulative no. of cases during study period^†^	773,512	22,455	45,490	528,440	39,132	14,112
Cumulative no. of deaths during study period^†^	11,934	445	291	7,345	721	499
Start of vaccination campaign	Feb. 7, 2021	Apr. 11, 2021	Mar. 1, 2021	Feb. 14, 2021	May 10, 2021	Mar. 31, 2021
Fully vaccinated individuals according to initial vaccination protocol by the end of study period (May 2021)^‡^	1.8%	0.38%	0.00%	3.29%	0.00%	0.00%
Most dominant circulating strains other than the wild-type during study period (up to May 4, 2021)^§^	Alpha and Beta	Alpha and Eta	Alpha and Eta	Alpha	Beta	Alpha and Eta

* Department of Economic and Social Affairs, United Nations. *World Population Prospects 2022*. https://population.un.org/wpp/Download/Standard/Population.

^†^ World Heath Organization. https://covid19.who.int/table.

^‡^ Our World in Data. https://ourworldindata.org/coronavirus#explore-the-global-situation.

^§^ CoVariants (data from GISAID). Covariants.org.

### Baseline demographic and clinical characteristics of hospitalized patients overall and by country.

**Figure 1.**
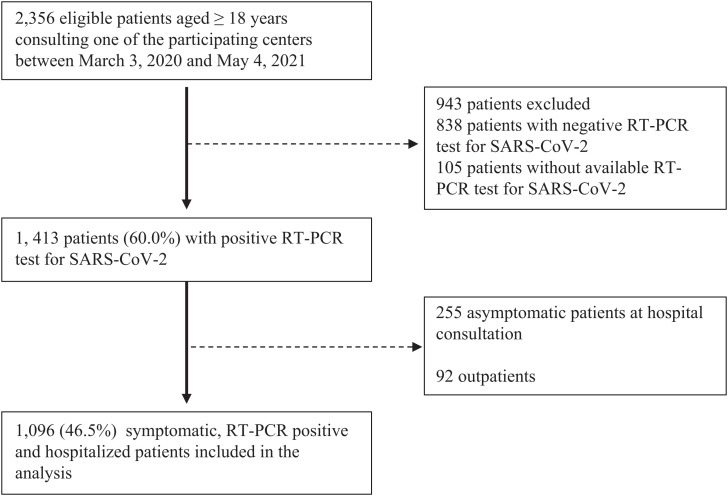
Study flow chart.

A flowchart depicting the procedure to select the study population is shown in Figure 1. The large majority of participating centers were university-affiliated hospitals. Of the 2,356 patients consulting one of the participating centers between March 3, 2020 and May 4, 2021, 1,413 patients (60.0%) with a positive RT-PCR test for SARS-CoV-2 were eligible to be enrolled. A total of 225 patients (9.6%) were asymptomatic at hospital consultation, and 96 (4.1%) were not hospitalized and consequently excluded from the analysis, leaving 1,096 (46.5%) patients for the analysis.

Table 2 describes patient characteristics, underlying comorbidities, clinical symptoms at baseline, and patient outcomes overall and by country. Overall, 671 patients (61.4%) were male, and the median age was 49 years, ranging from 38 in Mali to 63 years in Guinea. In total, 550 patients (50.2%) had at least one underlying comorbidity, cardiovascular disease (including hypertension) being the most prevalent in all countries (31.8%), followed by diabetes (24.3%). Lebanon and Mali had the smallest number of patients with preexisting comorbidities (25.0% and 26.7%, respectively). The three most frequent symptoms overall were fever (74.0%), cough (67.0%), and dyspnea (48.2%). The highest rates of headache (51.5%) and anosmia (30.7%) were reported by patients in Mali. The large majority of patients (81.0%) had abnormal lung x-ray findings.

**Table 2:** Baseline demographic and clinical characteristics of the study population in participating countries

Characteristics	Bangladesh (*n* = 565)	Guinea (*n* = 63)	Ivory Coast (*n* = 134)	Lebanon (*n* = 32)	Madagascar (*n* = 201)	Mali (*n* = 101)	Total (*n* = 1,096)
Demographic characteristics							
Male, *n* (%)	350 (62.0)	37 (58.7)	88 (65.7)	20 (62.5)	114 (62.5) [198]	62 (61.4)	671 (61.4) [1,093]
Sex ratio	1.6	1.4	1.9	1.7	1.4	1.6	1.6
Age, median (IQR)	46.0 (35.0–57.0)	63.0 (53.0–70.0)	55.0 (46.0–64.0)	55.5 (40.5–72.0)	51.0 (38.0–61.0) [197]	38.0 (28.0–51.0)	49.0 (36.0–60.0) [1,092]
Underlying comorbidities, *n* (%)	299 (52.9)	34 (54.0)	76 (56.7)	8 (25.0)	106 (52.7)	27 (26.7)	550 (50.2)
CVD including HTN	169 (29.9)	22 (34.9)	54 (40.3)	6 (18.8)	83 (41.3)	14 (13.9)	348 (31.8)
Diabetes	172 (30.4)	11 (17.5)	29 (21.6)	3 (9.4)	42 (20.9)	9 (8.9)	266 (24.3)
Chronic lung disease	41 (7.3)	8 (12.7)	8 (6.0)	2 (6.3)	5 (2.5)	4 (4.0)	68 (6.2)
Renal disease	21 (3.7)	1 (1.6)	3 (2.2)	0 (0.0)	6 (3.0)	0 (0.0)	31 (2.8)
Liver disease	10 (1.8)	0 (0.0)	0 (0.0)	0 (0.0)	1 (0.5)	1 (1.0)	12 (1.1)
Immuno-deficiency and HIV infection	1 (0.2)	1 (1.6)	1 (0.7)	0 (0.0)	3 (1.5)	1 (0.9)	7 (0.6)
Malignancy	2 (0.4)	0 (0.0)	0 (0.0)	0 (0.0)	1 (0.5)	1 (1.0)	4 (0.4)
Symptoms at admission, *n* (%)							
Fever	448 (79.3)	60 (95.2)	115 (85.8)	24 (75.0)	122 (60.7)	42 (41.6)	811 (74.0)
Cough	341 (60.4)	61 (96.8)	102 (76.1)	24 (75.0)	154 (76.6)	52 (51.5)	734 (67.0)
Dyspnea	272 (48.1)	52 (82.5)	72 (53.7)	16 (50.0)	98 (48.8)	18 (17.8)	528 (48.2)
Weakness	139 (24.6)	59 (93.7)	83 (61.9)	23 (71.9)	110 (54.7)	27 (26.7)	441 (40.2)
Pain	143 (25.3)	56 (88.9)	58 (43.3)	1 (3.1)	108 (53.7)	25 (24.8)	391 (35.7)
Chest pain	69 (12.2)	52 (82.5)	25 (18.7)	0 (0.0)	47 (23.4)	12 (11.9)	205 (18.7)
Myalgia	61 (10.8)	14 (22.2)	35 (26.1)	1 (3.1)	73 (36.3)	16 (15.8)	200 (18.2)
Abdominal pain	28 (5.0)	5 (7.9)	3 (2.2)	0 (0.0)	14 (7.0)	5 (5.0)	55 (5.0)
Joint pain	10 (1.8)	2 (3.2)	5 (3.7)	0 (0.0)	23 (11.4)	7 (6.9)	47 (4.3)
Headache	116 (20.5)	22 (34.9)	52 (38.8)	7 (21.9)	72 (35.8)	52 (51.5)	321 (29.3)
Anosmia	171 (30.3)	10 (15.9)	14 (10.5) [133]	0 (0.0)	29 (14.4)	31 (30.7)	255 (23.3) [1,095]
Sore throat	130 (23.0)	44 (69.8)	20 (14.9)	9 (28.1)	10 (5.0)	22 (21.8)	235 (21.4)
Runny nose	68 (12.0)	3 (4.8)	24 (17.9)	6 (18.8)	42 (20.9)	29 (28.7)	172 (15.7)
Ageusia	91 (16.2) [563]	26 (41.3)	16 (11.9)	0 (0.0)	9 (4.5)	24 (24.2) [99]	166 (15.2) [1,092]
Diarrhea	78 (13.8)	3 (4.8)	18 (13.4)	3 (9.4)	46 (22.9)	11 (10.9)	159 (14.5)
Nausea	53 (9.4)	2 (3.2)	20 (14.9)	2 (6.3)	15 (7.5)	14 (13.9)	106 (9.7)
Conjunctivitis	35 (6.2)	0 (0.0)	0 (0.0)	0 (0.0)	0 (0.0)	1 (1.0)	36 (3.3)
Confusion	11 (2.0)	2 (3.2)	3 (2.2)	2 (6.3)	10 (5.0)	4 (4.0)	32 (2.9)
Minor symptoms	18 (3.2)	1 (1.6)	1 (0.8)	0 (0.0)	8 (4.0)	17 (16.8)	45 (4.1)
Major symptoms	548 (96.8)	62 (98.4)	133 (99.3)	32 (100.0)	193 (96.0)	84 (83.2)	1,051 (95.9)
Median temp. at admission, °C (IQR)	38.8 (38.1–39.4) [320]	37.4 (37.2–38.0) [62]	37.5 (37.0–38.2) [95]	38.0 (37.0–38.5) [10]	36.9 (36.6–37.9)	36.8 (36.5–37.3) [96]	37.8 (36.9–38.8) [784]
Imaging test, *n* (%)							
Had a lung x-ray	182 (32.2)	59 (93.7)	100 (74.6)	5 (15.6)	94 (46.7)	9 (8.9)	449 (41.0)
Lung x-ray abnormal finding	117 (64.3)	51 (86.4)	96 (96.0)	2 (40.0)	89 (94.7)	8 (100.0) [8]	363 (81.0) [448]
Clinical severity, *n* (%)	50 (8.9) [560]	11 (17.5)	18 (15.7) [115]	5 (15.6)	43 (21.5) [200]	5 (6.7) [75]	134 (12.6) [1,045]
Admission to ICU, *n* (%)	14 (2.5) [560]	3 (4.8)	10 (8.7) [115]	5 (15.6)	40 (20.0) [200]	5 (6.7) [75]	77 (7.4) [1,045]
Death, *n* (%)	42 (7.4)	8 (12.7)	12 (9.0)	1 (3.1)	13 (6.5)	0 (0.0)	76 (6.9)
Median age of death in years, (IQR)	60.0 (51.0–67.0)	67.0 (54.5–71.0)	68.0 (64.0–73.0)	78.0	64.0 (50.0–71.0)	NA	64.5 (54.5–70.5)
Median delay between symptom onset and hospital admission (days) (IQR)	8.0 (6.0–11.0) [493]	15.0 (8.0–17.0) [14]	5.0 (1.0–9.0) [126]	5.0 (2.0–11.0) [18]	9.0 (6.0–14.0) [178]	5.0 (4.0–8.0) [84]	8.0 (5.0–11.0) [913]
Median duration of symptoms (IQR)	21.0 (16.0–27.0) [270]	21.0 (17.0–28.0) [43]	15.5 (10.5–26.5) [76]	13.0 (3.0–15.0) [3]	17.0 (12.0–26.0) [161]	9.0 (5.0–14.00) [62]	18.0 (13.0–26.0) [615]
Median duration of hospitalization (IQR)	15.0 (9.0–20.0) [565]	11.0 (4.0–19.0) [15]	10.0 (7.0–14.0) [128]	6.5 (4.0–14.5)	15.0 (10.0–25.0) [189]	NA	14.0 (9.0–20.0) [929]

CVD = cardiovascular disease; HTN = hypertension; ICU = intensive care unit; NA = not available; temp. = temperature. Data are presented as *n* (%) unless otherwise indicated. Median and interquartile ranges are indicated as median (IQR) when applicable. Data in square brackets indicate number of data available for the variable. If no square brackets, there are no missing data for the variable.

Of the 134 (12.6%) patients considered as clinically severe, 77 were admitted to the ICU, and 76 died during their hospitalization. The overall mortality rate was 6.9%, ranging from 0.0% in Mali to 12.7% in Guinea.

Median age at death in the study population was 64.5 years old and reached 78.0 years old in Lebanon. The median duration of symptoms was 18.0 days and varied from 9.0 days in Mali to 21.0 days in Bangladesh and Guinea. Patients were hospitalized for a median duration of 14.0 days ranging from 6.5 days in Lebanon to 15.0 days in Bangladesh and Madagascar.

### Baseline demographic and clinical characteristics of patients by age group and clinical severity.

Table 3 describes the main characteristics of the patients according to age group and clinical severity. The proportion of patients with underlying comorbidities, presence of major symptoms, or clinical severity was significantly higher in older patients ≥60 years old. Patients aged <60 years were more frequently female, and duration of their symptoms was shorter than those ≥60 years (18.0 days versus 20.0 days, respectively, *P* = 0.004). We did not observe any statistically significant differences in the duration of hospitalization between the two age groups (15.0 days in <60 years old and 14.0 days in ≥60 years old, *P* = 0.47).

**Table 3:** Baseline demographic and clinical characteristics of the study population by age category and by clinical severity

Characteristic	Age <60 (*n* = 810)	Age ≥ 60 (*n* = 282)	*P*-Value	Patients Admitted to Medical Ward (*n* = 962)	Patients Admitted to ICU and/or Death during Hospitalization (patients with clinical severity) (*n* = 134)	*P*-Value
Demographics						
Male sex, *n* (%)	481 (59.5) [808]	187 (66.6)	0.04	543 (59.5)	94 (71.8)	0.007
Sex ratio	1.5	2.0		1.5	2.5	
Age, median (IQR)	42.0 (32.0–51.0)	66.0 (63.0–71.0)	<0.001	46.0 (35.0–58.0) [910]	61.0 (54.0–69.0) [133]	<0.001
Underlying comorbidities, *n* (%)	341 (42.1)	207 (73.4)	<0.001	428 (46.9)	101 (75.4)	<0.001
CVD including HTN	188 (23.2)	159 (56.4)	<0.001	262 (28.7)	78 (58.2)	<0.001
Diabetes	169 (20.9)	97 (34.4)	<0.001	211 (23.1)	45 (33.6)	0.008
Chronic lung disease	43 (5.3)	25 (8.9)	0.03	58 (6.4)	8 (6.0)	0.87
Renal disease	20 (2.5)	11 (3.9)	0.21	22 (2.4)	8 (6.0)	0.02
Liver disease	8 (1.0)	4 (1.4)	0.52	9 (1.0)	2 (1.5)	0.64
Immuno-deficiency and HIV	5 (0.6)	1 (0.4)	0.99	4 (0.9)	0 (0.0)	0.99
Malignancy	2 (0.2)	2 (0.7)	0.28	3 (0.3)	1 (0.8)	0.42
Symptoms at admission, *n* (%)						
Fever	594 (73.3)	214 (75.9)	0.40	666 (73.0)	119 (88.8)	<0.001
Cough	522 (64.4)	209 (74.1)	0.003	588 (64.4)	113 (84.3)	<0.001
Dyspnea	356 (44.0)	170 (60.3)	<0.001	402 (44.0)	104 (77.6)	<0.001
Weakness	296 (36.5)	144 (51.1)	<0.001	339 (37.1)	88 (65.7)	<0.001
Pain	272 (33.6)	116 (41.1)	0.02	301 (33.0)	77 (57.5)	<0.001
Chest pain	130 (16.1)	74 (26.2)	<0.001	160 (17.5)	39 (29.1)	0.001
Myalgia	144 (17.8)	55 (19.5)	0.52	143 (15.7)	49 (36.6)	<0.001
Abdominal pain	43 (5.3)	11 (3.9)	0.35	46 (5.0)	8 (6.0)	0.65
Joint pain	37 (4.6)	10 (3.6)	0.47	38 (4.2)	6 (4.5)	0.87
Headache	238 (29.4)	80 (28.4)	0.75	253 (27.7)	54 (40.3)	0.003
Anosmia	191 (23.6)	63 (22.4) [281]	0.69	213 (23.4) [912]	32 (23.9)	0.89
Sore throat	178 (22.0)	57 (20.2)	0.54	205 (22.5)	19 (14.2)	0.03
Runny nose	141 (17.4)	31 (11.0)	0.01	141 (15.4)	21 (15.7)	0.95
Ageusia	113 (14.0) [806]	53 (18.8)	0.06	146 (16.0) [911]	18 (13.4)	0.44
Diarrhea	124 (15.3)	34 (12.1)	0.18	131 (14.4)	23 (17.2)	0.39
Nausea	76 (9.4)	29 (10.3)	0.66	87 (9.5)	15 (11.2)	0.54
Conjunctivitis	30 (3.7)	6 (2.1)	0.20	29 (3.2)	6 (4.5)	0.43
Confusion	24 (3.0)	8 (2.8)	0.91	21 (2.3)	9 (6.7)	0.004
Major symptoms	42 (5.2)	3 (1.1)	0.003	870 (95.3)	133 (99.3)	0.03
Minor symptoms	768 (94.8)	279 (98.9)	0.003	43 (4.7)	1 (0.8)	0.03
Median temp. at admission, °C (IQR)	37.8 (36.9–38.8) [575]	37.8 (37.0–38.8) [205]	0.97	37.8 (37.0–38.8) [626]	38.0 (36.9–38.9) [114]	0.22
Imaging test, *n* (%)						
Had a lung x-ray	290 (35.8)	158 (56.0)	<0.001	362 (39.7)	68 (50.8)	0.01
Lung x-ray abnormal finding	223 (77.2) [289]	139 (88.0)	0.004	280 (77.3)	66 (97.1)	<0.001
Clinical severity, *n* (%)	58 (7.5) [770]	73 (26.9) [271]	<0.001	0 (0.0)	132 (100.0)	<0.001
Admission to ICU, *n* (%)	40 (5.2) [770]	36 (13.3) [271]	<0.001	0 (0.0)	77 (58.3)	<0.001
Death, *n* (%)	27 (3.3)	49 (17.4)	<0.001	0 (0.0)	76 (56.7)	<0.001
Median age of death (years) (IQR)	50.0 (38.0–55.0)	69.0 (65.0–74.0)	<0.001	NA	64.5 (54.5–70.5)	NA
Median delay between symptom onset and hospital admission (days) (IQR)	8.0 (5.0–11.0) [691]	8.0 (5.0–11.0) [218]	0.14	8.0 (5.0–11.0) [781]	8.0 (5.0–9.0) [114]	0.31
Median duration of symptoms (IQR)	18.0 (12.0–25.0) [459]	20.0 (15.0–28.0) [152]	0.004	19.0 (13.0–26.0) [530]	22.0 (11.0–32.0) [59]	0.20
Median duration of hospitalization (IQR)	15.0 (9.0–21.0) [698]	14.0 (8.0–20.0) [227]	0.47	15.0 (9.0–20.0) [793]	10.0 (5.0–21.0) [114]	0.01

CVD = cardiovascular disease; HTN = hypertension; ICU = intensive care unit; NA = not available; temp = temperature.

Data are presented as *n* (%) unless otherwise indicated median and interquartile ranges are indicated as median (IQR) when applicable. Data in square brackets indicate number of data available for the variable. If no square brackets, there are no missing data for the variable. Minor symptoms: headache, anosmia, ageusia, sore throat, runny nose, diarrhea, nausea, conjunctivitis. Major symptoms: fever, cough, dyspnea, pain, weakness, confusion.

Compared with patients hospitalized in medical wards, those identified as clinically severe were more frequently male, older, and reported as more frequently having underlying comorbidities and major symptoms.

### Predictors of clinical severity.

Multivariable logistic regression models showed that in patients <60 years old, presence of cardiovascular disease (adjusted odds ratio [aOR]: 2.47; 95% CI: 1.33–4.57, *P* = 0.004) and diabetes (aOR: 2.13; 95% CI: 1.11–4.10, *P* = 0.02) were associated with the risk of clinical severity (Table 4). In patients ≥60 years, only cardiovascular disease (aOR: 1.99; 95% CI: 1.13–3.50, *P* = 0.018) was independently associated with the risk of severe outcome (Table 4).

**Table 4:** Multivariable logistic regression of factors associated with risk of clinical severity

Factor	Patients Aged <60 years				Patients Aged ≥60 years			
	Univariate Analysis		Multivariable Analysis*		Univariate Analysis		Multivariable Analysis*	
	OR (95% CI)	*P*-Value	aOR (95% CI)	*P*-Value	OR (95% CI)	*P*-Value	aOR (95% CI)	*P*-Value
Male sex	1.76 (0.97–3.20)	0.06	1.69 (0.91–3.14)	0.10	1.30 (0.73–2.33)	0.37	1.36 (0.75–2.44)	0.31
Comorbidities								
CVD	3.20 (1.85–5.51)	<0.001	2.47 (1.33–4.57)	0.004	1.99 (1.14–3.50)	0.02	1.99 (1.13–3.50)	0.02
Diabetes	2.52 (1.44–4.40)	0.001	2.13 (1.11–4.10)	0.02	0.77 (0.43–1.37)	0.37	–	–
Chronic lung disease	1.56 (0.54–4.57)	0.41	–	–	0.32 (0.07–1.41)	0.13	–	–
Renal disease	2.35 (0.67–8.29)	0.18	–	–	1.96 (0.54–7.16)	0.31	–	–
Liver disease	2.18 (0.26–18.43)	0.47	–	–	0.95 (0.10–9.31)	0.97	–	–

aOR = adjusted odds ratio; CVD = cardiovascular disease; OR = odds ratio.

* Multivariable logistic regression with a random effect on country. Models were adjusted on sex and any variable with *P* <0.20 in univariate analysis.

## DISCUSSION

To the best of our knowledge, this prospective hospital-based study is one of the few to investigate the clinical characteristics and outcomes of COVID-19 patients in several LMICs using a standard methodology. The study included 1,096 laboratory-confirmed COVID-19 symptomatic patients.

The gender propensity was similar to what has been observed in other studies carried out in the region^15^^,^^16^ or in other parts of the world.^3^ As in other studies, male gender tends to be a severity risk factor, particularly in the younger age group, even if the association did not reach statistical significance.^17^ Genetic parameters, sex hormones, immune system responses, and other nonbiological causes have been proposed to explain the sex disparity, but their actual role remains unclear.^18^ This is in agreement with another study reporting that at equal age, males with COVID-19 are at higher risk of worse outcomes and death than females.^19^

The most frequently reported symptoms at admission (fever, cough, and dyspnea) in this relatively young population were similar to what has been observed among older populations in developed countries.^20^^,^^21^ As expected, the number of underlying comorbidities as well as disease severity were significantly higher among patients ≥60 years old.

Older age and underlying diseases are known factors of COVID-19-related mortality.^3^ The overall mortality rate during the study period among the included participants was 6.9%, ranging from 0.0% to 14.1%, much lower than what we have observed in the French population of the NOSO-COR study (17.9%, unpublished data) or in other high-income countries.^22^ Mortality rate was the highest in Guinea (14.1%), whereas no death occurred among the study patients in Mali. The ICU mortality rate was 25% in a cross-sectional study carried out at the early stage of the pandemic in Guinea.^23^ This higher mortality rate could be explained by the much older age of patients from Guinea as well as the higher number of existing comorbidities in this study population compared with those from Mali. Bangladesh had the second lowest mortality rates after Mali, most probably due to demographic characteristics, with approximately 80% of the country population being <50 years old. Similar results were reported in another study.^24^

Having underlying cardiovascular disease has been shown to be associated with COVID-19 severity.^25^ We also found a positive association between preexisting cardiovascular diseases and clinical severity in all patients. Moreover, the risk was higher in younger patients aged <60 years, suggesting that an altered general state with underlying conditions is a risk factor of clinical severity in younger patients.

At the early course of the SARS-CoV-2 pandemic, it was hypothesized that due to relatively limited air traffic with China, the virus was less likely to enter Francophone West African countries.^26^ However, the first cases of COVID-19 were detected in all participating countries a few days before or after the declaration of the pandemic by the WHO. The first identified cases were all travelers returning from Europe^27^^,^^28^ except for Lebanon, which identified its first case of COVID-19 in a woman traveling back from pilgrimage in Iran.^29^

Despite the almost simultaneous arrival of the pandemic in all participating countries, COVID-19 incidence has evolved differently, with some countries showing an exponential rise in incident cases and others a gradual and linear evolution.^27^^,^^30^

Of the participating countries, four (Bangladesh, Lebanon, Mali, Madagascar) are part of the Mérieux Foundation GABRIEL Network (https://www.gabriel-network.org/), which has extensive experience in conducting research on viral respiratory diseases. The use of the same questionnaire and methodology is another strength of the present study. In addition, sample size was large enough to be able to assess risk factors independently associated with severe outcome. The study was carried out during the surge of the pandemic in countries with several issues that could slow down the research. Despite limited availability of personal protective equipment, all centers carried patient interviews to record the study-related information, thus increasing the reliability of the collected data.

Our study does present some limitations, however. One was possible selection bias. In addition, the complexity of case definition criteria, testing strategies, and availability of tests in LMICs, particularly at the beginning of the study, could influence the inclusion flow. To preserve the quality of healthcare systems parallel to the exponential increase in the number of COVID-19 patients at the early phases of the pandemic, patient criteria for hospitalization might also have changed. This could lead to more severe hospitalized cases. Also, due to the emergency situation of the pandemic during the study period, some confounding variables, such as medication and vital signs, during hospitalization could not be collected and taken into consideration for the statistical analyses.

Finally, different viral strains were spreading in the participating centers during the study period, and it is well known that clinical severity may differ according to the strains. However, because of the lack of appropriate laboratory facilities in the participating countries, information on the circulating strains was not available.

In conclusion, although the existence of different care practices between countries makes the comparison difficult, our findings are consistent with those described elsewhere, suggesting that COVID-19-related severity and death are mainly driven by older age.

Further studies are needed to assess whether the organization and quality of healthcare systems in LMICs, and particularly the access to tertiary care, could have had an impact on COVID-19 clinical outcomes.

## Supplemental Materials

10.4269/ajtmh.23-0456 Supplemental Materials
