# Supplementary material for: Characteristics of Hospitalized COVID-19 Patients at Admission and Factors Associated with Clinical Severity in Low- and Middle-Income Countries: An Observational Study
Source: Am J Trop Med Hyg. 2024 Feb 27;110(4):741–8. doi: 10.4269/ajtmh.23-0456 (PMC10993839; doi:10.4269/ajtmh.23-0456)

Supplementary Material

Table S1. Description of sites, dates of ethical approval, first and last patient inclusion by country

**Country**	**Centres**	**Date of approval**	**Name of the committee**	**Date of first patient inclusion**	**Date of last patient inclusion**
Bangladesh	3 centers<br>Chittagong Medical College Hospital, Chittagong<br>Khulna Medical College Hospital, Khulna<br>Mugda Medical College Hospital, Dhaka	April 2, 2020<br>April 10, 2020	Research Review Committee<br>Ethical review Committee	May 2, 2020	August 26, 2020
Guinea	2 centers<br>Hôpital National Donka, Conakry<br>Hôpital National Ignace Deen, Conakry	April 20, 2020	Comité National d'Ethique pour la Recherche en Santé	May 30, 2020	April 16, 2021
Ivory Coast	5 centers<br>Centre Hospitalier et Universitaire d'Angré, Abidjan<br>Hôpital Général d'Anyama, Abidjan<br>Centre Hospitalier Universitaire de Cocody, Abidjan<br>Polyclinique Farah, Marcory, Abidjan<br>Hôpital Militaire d'Abidjan, Abidjan	April 10, 2020	Comité National d'Ethique des Sciences de la Vie et de la Santé	May 3, 2020	March 31, 2021
Lebanon	1 center<br>Hôpital Hôtel-Dieu de France, Beirut	March 5, 2020	Comité d'éthique de l'Hôtel Dieu de France	March 5, 2020	September 23, 2020
Mali	2 centers<br>Centre Hospitalier Universitaire du Point G, Bamako<br>Hôpital du Mali, Bamako	April 16, 2020	Comité d'Ethique de la Faculté de Médecine et Odonto-Stomatologie et Faculté de Pharmacie	May 8, 2020	December 4, 2020

Madagascar	3 centers<br>Centre Hospitalier Universitaire Befelatanana, Antananarivo<br>Centre Hospitalier Universitaire Andohatapenaka, Antananarivo<br>Centre Hospitalier Universitaire Aniosala, Alakamisy Ambohidratrimo	March 30, 2020	Comité d'Ethique de la Recherche Biomédicale	May 1, 2020	May 4, 2021

Supplementary Figure S1: **Map of NOSO-COR participating centers**

The figure illustrates all participating countries and the number of centers per country.

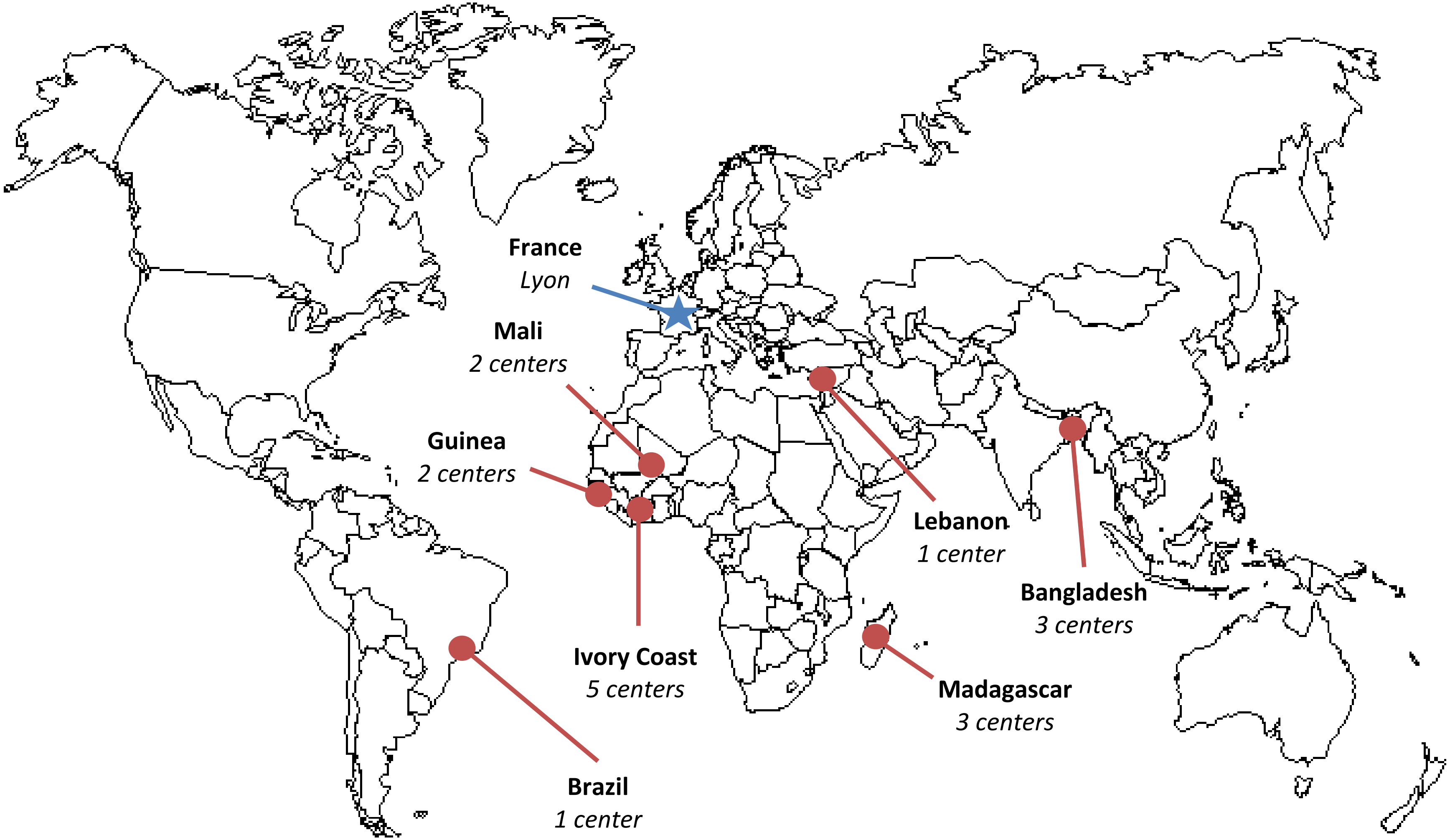

Supplement: Supplemental Materials [file tpmd230456.SD1.pdf]
